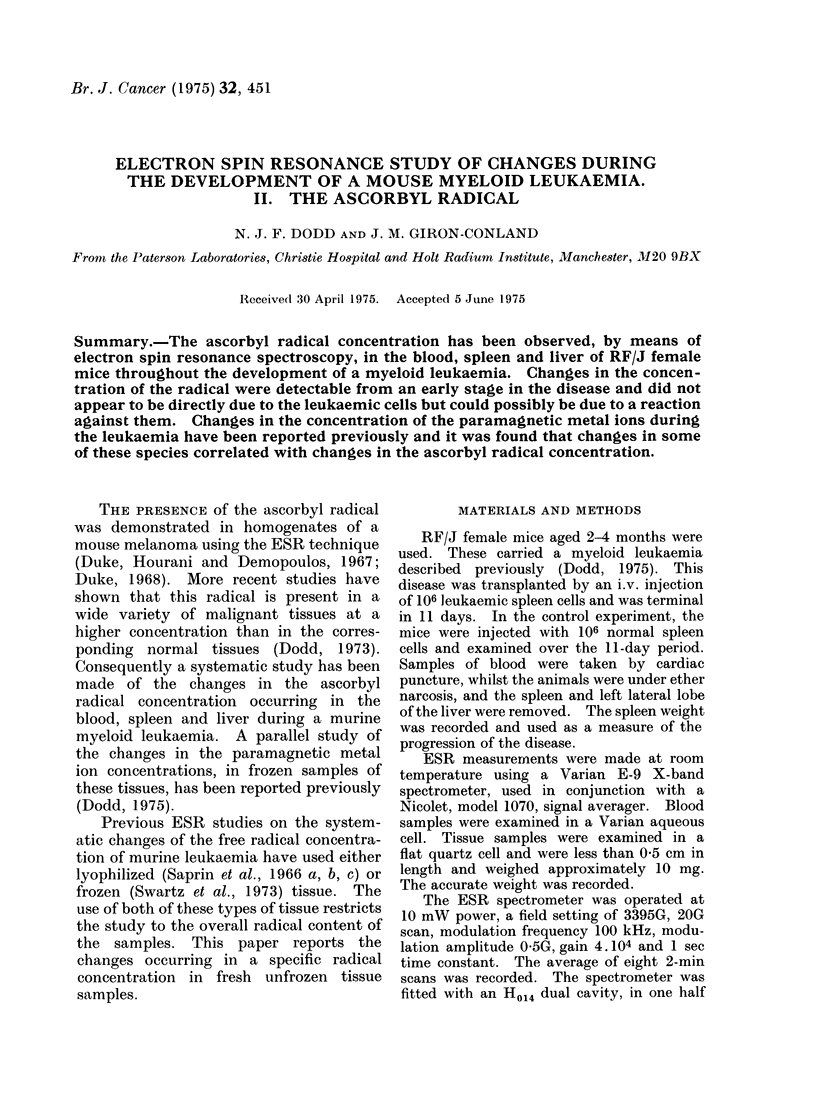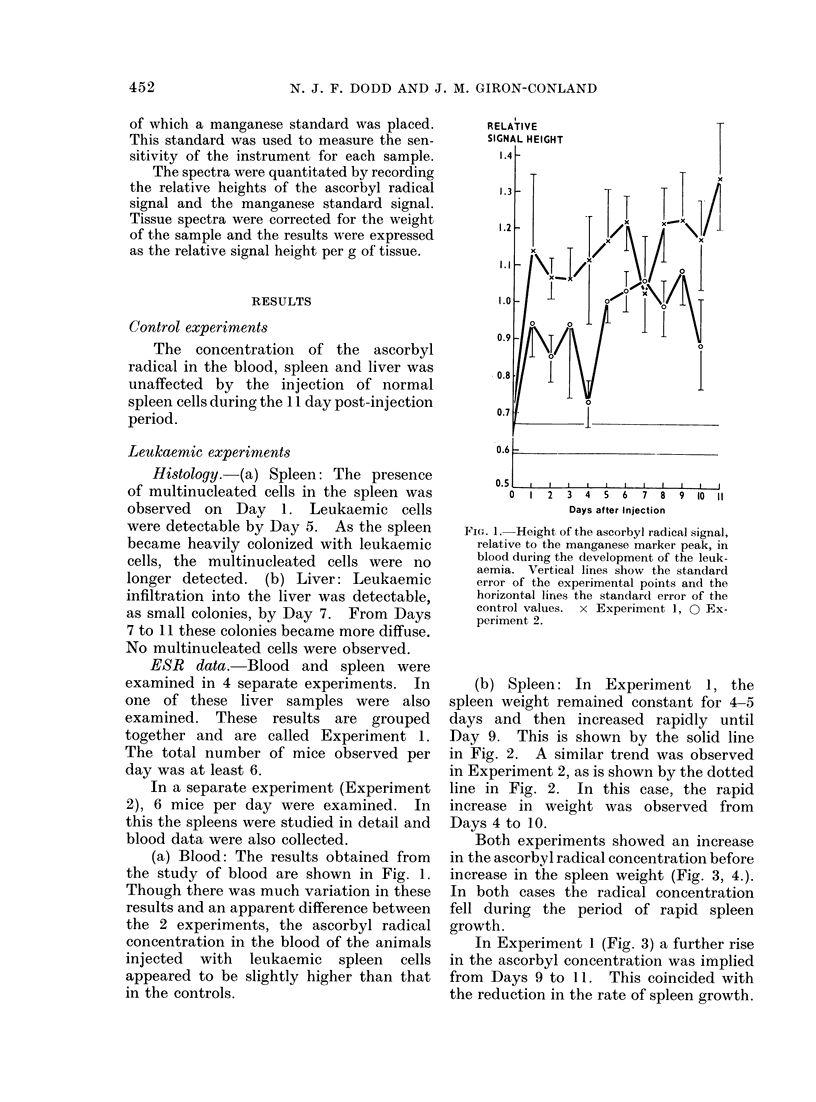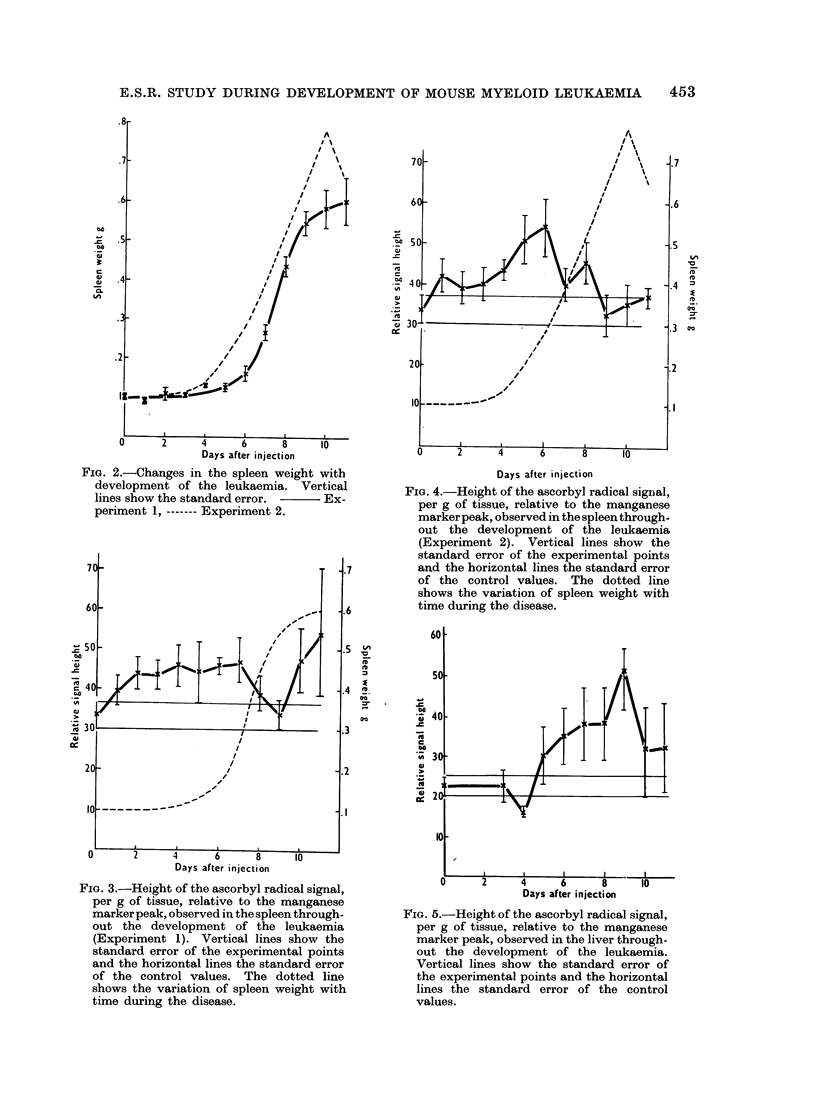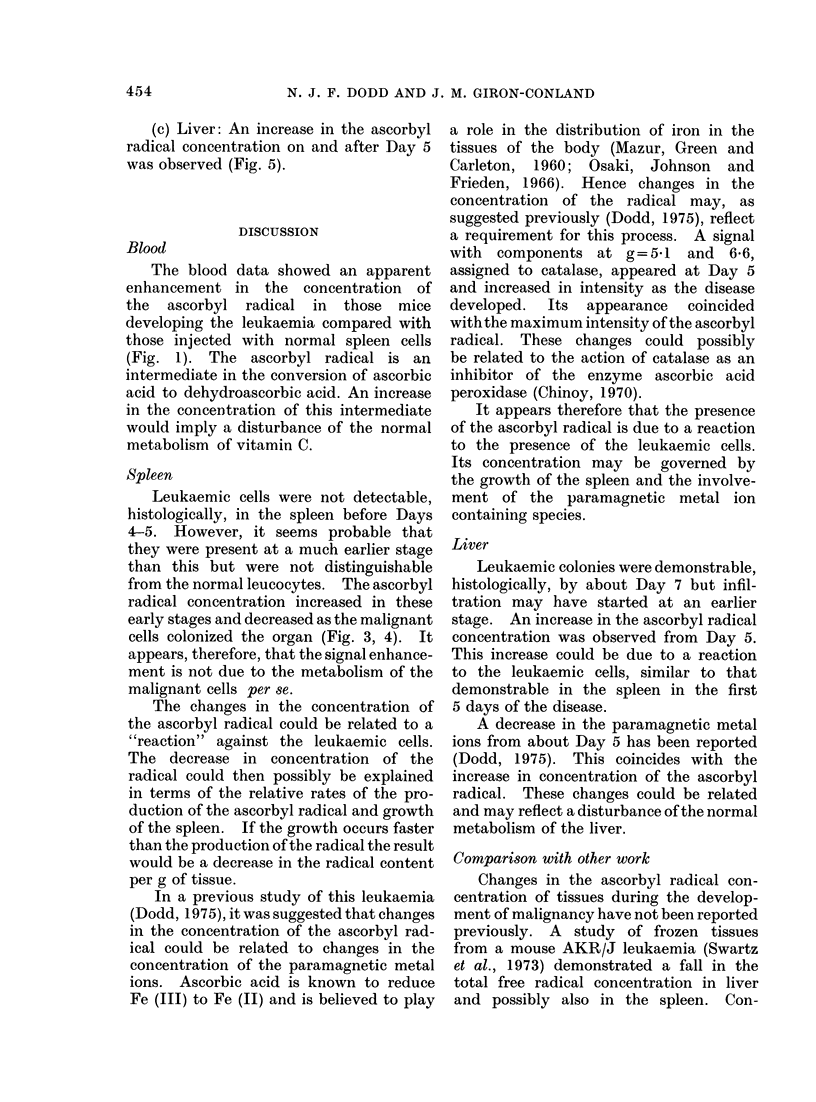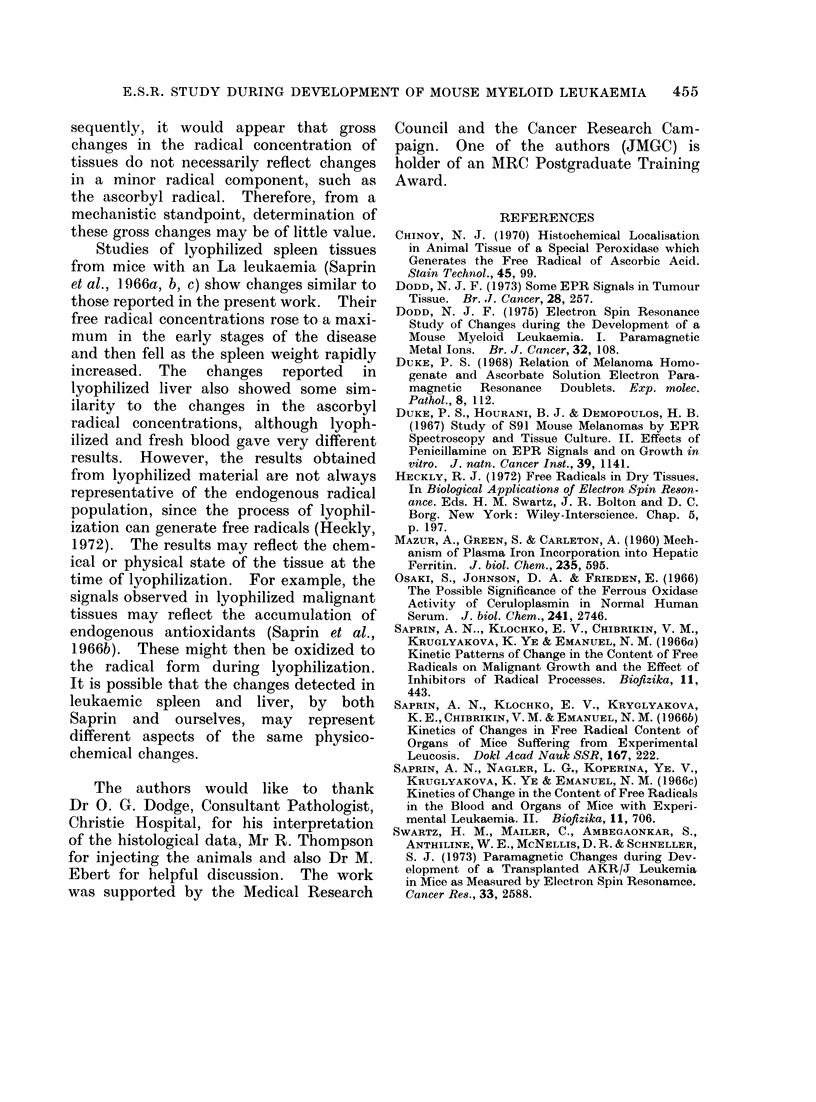# Electron spin resonance study of changes during the development of a mouse myeloid leukaemia. II. The ascorbyl radical.

**DOI:** 10.1038/bjc.1975.246

**Published:** 1975-10

**Authors:** N. J. Dodd, J. M. Giron-Conland

## Abstract

The ascorbyl radical concentration has been observed, by means of electron spin resonance spectroscopy, in the blood, spleen and liver of RF/J female mice throughout the development of a myeloid leukaemia. Changes in the concentration of the radical were detectable from an early stage in the disease and did not appear to be directly due to the leukaemic cells but could possibly be due to a reaction against them. Changes in the concentration of the paramagnetic metal ions during the leukaemia have been reported previously and it was found that changes in some of these species correlated with changes in the ascorbyl radical concentration.


*Br. J. Cancer* (1975) **32**, 451

### ELECTRON SPIN RESONANCE STUDY OF CHANGES DURING THE DEVELOPMENT OF A MOUSE MYELOID LEUKAEMIA. II. THE ASCORBYL RADICAL

N. J. F. DODD AND J. M. GIRON-CONLAND

*From the Paterson Laboratories, Christie Hospital and Holt Radium Institute, Manchester, M20 9BX*

Received 30 April 1975. Accepted 5 June 1975

**Summary.—The ascorbyl radical concentration has been observed, by means of electron spin resonance spectroscopy, in the blood, spleen and liver of RF/J female mice throughout the development of a myeloid leukaemia. Changes in the concentration of the radical were detectable from an early stage in the disease and did not appear to be directly due to the leukaemic cells but could possibly be due to a reaction against them. Changes in the concentration of the paramagnetic metal ions during the leukaemia have been reported previously and it was found that changes in some of these species correlated with changes in the ascorbyl radical concentration.**

THE PRESENCE of the ascorbyl radical was demonstrated in homogenates of a mouse melanoma using the ESR technique (Duke, Hourani and Demopoulos, 1967; Duke, 1968). More recent studies have shown that this radical is present in a wide variety of malignant tissues at a higher concentration than in the corresponding normal tissues (Dodd, 1973). Consequently a systematic study has been made of the changes in the ascorbyl radical concentration occurring in the blood, spleen and liver during a murine myeloid leukaemia. A parallel study of the changes in the paramagnetic metal ion concentrations, in frozen samples of these tissues, has been reported previously (Dodd, 1975).

Previous ESR studies on the systematic changes of the free radical concentration of murine leukaemia have used either lyophilized (Saprin *et al.*, 1966 *a*, *b*, *c*) or frozen (Swartz *et al.*, 1973) tissue. The use of both of these types of tissue restricts the study to the overall radical content of the samples. This paper reports the changes occurring in a specific radical concentration in fresh unfrozen tissue samples.

### MATERIALS AND METHODS

RF/J female mice aged 2–4 months were used. These carried a myeloid leukaemia described previously (Dodd, 1975). This disease was transplanted by an i.v. injection of $10^6$ leukaemic spleen cells and was terminal in 11 days. In the control experiment, the mice were injected with $10^6$ normal spleen cells and examined over the 11-day period. Samples of blood were taken by cardiac puncture, whilst the animals were under ether narcosis, and the spleen and left lateral lobe of the liver were removed. The spleen weight was recorded and used as a measure of the progression of the disease.

ESR measurements were made at room temperature using a Varian E-9 X-band spectrometer, used in conjunction with a Nicolet, model 1070, signal averager. Blood samples were examined in a Varian aqueous cell. Tissue samples were examined in a flat quartz cell and were less than 0·5 cm in length and weighed approximately 10 mg. The accurate weight was recorded.

The ESR spectrometer was operated at 10 mW power, a field setting of 3395G, 20G scan, modulation frequency 100 kHz, modulation amplitude 0·5G, gain $4.10^4$ and 1 sec time constant. The average of eight 2-min scans was recorded. The spectrometer was fitted with an $H_{014}$ dual cavity, in one half of which a manganese standard was placed. This standard was used to measure the sensitivity of the instrument for each sample.

The spectra were quantitated by recording the relative heights of the ascorbyl radical signal and the manganese standard signal. Tissue spectra were corrected for the weight of the sample and the results were expressed as the relative signal height per g of tissue.

### RESULTS

### *Control experiments*

The concentration of the ascorbyl radical in the blood, spleen and liver was unaffected by the injection of normal spleen cells during the 11 day post-injection period.

### *Leukaemic experiments*

*Histology.*—(a) Spleen: The presence of multinucleated cells in the spleen was observed on Day 1. Leukaemic cells were detectable by Day 5. As the spleen became heavily colonized with leukaemic cells, the multinucleated cells were no longer detected. (b) Liver: Leukaemic infiltration into the liver was detectable, as small colonies, by Day 7. From Days 7 to 11 these colonies became more diffuse. No multinucleated cells were observed.

*ESR data.*—Blood and spleen were examined in 4 separate experiments. In one of these liver samples were also examined. These results are grouped together and are called Experiment 1. The total number of mice observed per day was at least 6.

In a separate experiment (Experiment 2), 6 mice per day were examined. In this the spleens were studied in detail and blood data were also collected.

(a) Blood: The results obtained from the study of blood are shown in Fig. 1. Though there was much variation in these results and an apparent difference between the 2 experiments, the ascorbyl radical concentration in the blood of the animals injected with leukaemic spleen cells appeared to be slightly higher than that in the controls.

FIG. 1.—Height of the ascorbyl radical signal, relative to the manganese marker peak, in blood during the development of the leukaemia. Vertical lines show the standard error of the experimental points and the horizontal lines the standard error of the control values. × Experiment 1, ○ Experiment 2.

(b) Spleen: In Experiment 1, the spleen weight remained constant for 4–5 days and then increased rapidly until Day 9. This is shown by the solid line in Fig. 2. A similar trend was observed in Experiment 2, as is shown by the dotted line in Fig. 2. In this case, the rapid increase in weight was observed from Days 4 to 10.

Both experiments showed an increase in the ascorbyl radical concentration before increase in the spleen weight (Fig. 3, 4.). In both cases the radical concentration fell during the period of rapid spleen growth.

In Experiment 1 (Fig. 3) a further rise in the ascorbyl concentration was implied from Days 9 to 11. This coincided with the reduction in the rate of spleen growth.

FIG. 2.—Changes in the spleen weight with development of the leukaemia. Vertical lines show the standard error. ——— Experiment 1, ------ Experiment 2.

FIG. 3.—Height of the ascorbyl radical signal, per g of tissue, relative to the manganese marker peak, observed in the spleen throughout the development of the leukaemia (Experiment 1). Vertical lines show the standard error of the experimental points and the horizontal lines the standard error of the control values. The dotted line shows the variation of spleen weight with time during the disease.

FIG. 4.—Height of the ascorbyl radical signal, per g of tissue, relative to the manganese marker peak, observed in the spleen throughout the development of the leukaemia (Experiment 2). Vertical lines show the standard error of the experimental points and the horizontal lines the standard error of the control values. The dotted line shows the variation of spleen weight with time during the disease.

FIG. 5.—Height of the ascorbyl radical signal, per g of tissue, relative to the manganese marker peak, observed in the liver throughout the development of the leukaemia. Vertical lines show the standard error of the experimental points and the horizontal lines the standard error of the control values.

(c) Liver: An increase in the ascorbyl radical concentration on and after Day 5 was observed (Fig. 5).

### DISCUSSION

*Blood*

The blood data showed an apparent enhancement in the concentration of the ascorbyl radical in those mice developing the leukaemia compared with those injected with normal spleen cells (Fig. 1). The ascorbyl radical is an intermediate in the conversion of ascorbic acid to dehydroascorbic acid. An increase in the concentration of this intermediate would imply a disturbance of the normal metabolism of vitamin C.

*Spleen*

Leukaemic cells were not detectable, histologically, in the spleen before Days 4–5. However, it seems probable that they were present at a much earlier stage than this but were not distinguishable from the normal leucocytes. The ascorbyl radical concentration increased in these early stages and decreased as the malignant cells colonized the organ (Fig. 3, 4). It appears, therefore, that the signal enhancement is not due to the metabolism of the malignant cells *per se*.

The changes in the concentration of the ascorbyl radical could be related to a "reaction" against the leukaemic cells. The decrease in concentration of the radical could then possibly be explained in terms of the relative rates of the production of the ascorbyl radical and growth of the spleen. If the growth occurs faster than the production of the radical the result would be a decrease in the radical content per g of tissue.

In a previous study of this leukaemia (Dodd, 1975), it was suggested that changes in the concentration of the ascorbyl radical could be related to changes in the concentration of the paramagnetic metal ions. Ascorbic acid is known to reduce Fe (III) to Fe (II) and is believed to play a role in the distribution of iron in the tissues of the body (Mazur, Green and Carleton, 1960; Osaki, Johnson and Frieden, 1966). Hence changes in the concentration of the radical may, as suggested previously (Dodd, 1975), reflect a requirement for this process. A signal with components at $g=5{\cdot}1$ and $6{\cdot}6$, assigned to catalase, appeared at Day 5 and increased in intensity as the disease developed. Its appearance coincided with the maximum intensity of the ascorbyl radical. These changes could possibly be related to the action of catalase as an inhibitor of the enzyme ascorbic acid peroxidase (Chinoy, 1970).

It appears therefore that the presence of the ascorbyl radical is due to a reaction to the presence of the leukaemic cells. Its concentration may be governed by the growth of the spleen and the involvement of the paramagnetic metal ion containing species.

*Liver*

Leukaemic colonies were demonstrable, histologically, by about Day 7 but infiltration may have started at an earlier stage. An increase in the ascorbyl radical concentration was observed from Day 5. This increase could be due to a reaction to the leukaemic cells, similar to that demonstrable in the spleen in the first 5 days of the disease.

A decrease in the paramagnetic metal ions from about Day 5 has been reported (Dodd, 1975). This coincides with the increase in concentration of the ascorbyl radical. These changes could be related and may reflect a disturbance of the normal metabolism of the liver.

*Comparison with other work*

Changes in the ascorbyl radical concentration of tissues during the development of malignancy have not been reported previously. A study of frozen tissues from a mouse AKR/J leukaemia (Swartz *et al.*, 1973) demonstrated a fall in the total free radical concentration in liver and possibly also in the spleen. Consequently, it would appear that gross changes in the radical concentration of tissues do not necessarily reflect changes in a minor radical component, such as the ascorbyl radical. Therefore, from a mechanistic standpoint, determination of these gross changes may be of little value.

Studies of lyophilized spleen tissues from mice with an La leukaemia (Saprin *et al.*, 1966*a*, *b*, *c*) show changes similar to those reported in the present work. Their free radical concentrations rose to a maximum in the early stages of the disease and then fell as the spleen weight rapidly increased. The changes reported in lyophilized liver also showed some similarity to the changes in the ascorbyl radical concentrations, although lyophilized and fresh blood gave very different results. However, the results obtained from lyophilized material are not always representative of the endogenous radical population, since the process of lyophilization can generate free radicals (Heckly, 1972). The results may reflect the chemical or physical state of the tissue at the time of lyophilization. For example, the signals observed in lyophilized malignant tissues may reflect the accumulation of endogenous antioxidants (Saprin *et al.*, 1966*b*). These might then be oxidized to the radical form during lyophilization. It is possible that the changes detected in leukaemic spleen and liver, by both Saprin and ourselves, may represent different aspects of the same physicochemical changes.

The authors would like to thank Dr O. G. Dodge, Consultant Pathologist, Christie Hospital, for his interpretation of the histological data, Mr R. Thompson for injecting the animals and also Dr M. Ebert for helpful discussion. The work was supported by the Medical Research Council and the Cancer Research Campaign. One of the authors (JMGC) is holder of an MRC Postgraduate Training Award.

### REFERENCES

CHINOY, N. J. (1970) Histochemical Localisation in Animal Tissue of a Special Peroxidase which Generates the Free Radical of Ascorbic Acid. *Stain Technol.*, **45**, 99.

DODD, N. J. F. (1973) Some EPR Signals in Tumour Tissue. *Br. J. Cancer*, **28**, 257.

DODD, N. J. F. (1975) Electron Spin Resonance Study of Changes during the Development of a Mouse Myeloid Leukaemia. I. Paramagnetic Metal Ions. *Br. J. Cancer*, **32**, 108.

DUKE, P. S. (1968) Relation of Melanoma Homogenate and Ascorbate Solution Electron Paramagnetic Resonance Doublets. *Exp. molec. Pathol.*, **8**, 112.

DUKE, P. S., HOURANI, B. J. & DEMOPOULOS, H. B. (1967) Study of S91 Mouse Melanomas by EPR Spectroscopy and Tissue Culture. II. Effects of Penicillamine on EPR Signals and on Growth *in vitro*. *J. natn. Cancer Inst.*, **39**, 1141.

HECKLY, R. J. (1972) Free Radicals in Dry Tissues. In *Biological Applications of Electron Spin Resonance*. Eds. H. M. Swartz, J. R. Bolton and D. C. Borg. New York: Wiley-Interscience. Chap. 5, p. 197.

MAZUR, A., GREEN, S. & CARLETON, A. (1960) Mechanism of Plasma Iron Incorporation into Hepatic Ferritin. *J. biol. Chem.*, **235**, 595.

OSAKI, S., JOHNSON, D. A. & FRIEDEN, E. (1966) The Possible Significance of the Ferrous Oxidase Activity of Ceruloplasmin in Normal Human Serum. *J. biol. Chem.*, **241**, 2746.

SAPRIN, A. N., KLOCHKO, E. V., CHIBRIKIN, V. M., KRUGLYAKOVA, K. YE & EMANUEL, N. M. (1966*a*) Kinetic Patterns of Change in the Content of Free Radicals on Malignant Growth and the Effect of Inhibitors of Radical Processes. *Biofizika*, **11**, 443.

SAPRIN, A. N., KLOCHKO, E. V., KRYGLYAKOVA, K. E., CHIBRIKIN, V. M. & EMANUEL, N. M. (1966*b*) Kinetics of Changes in Free Radical Content of Organs of Mice Suffering from Experimental Leucosis. *Dokl Acad Nauk SSR*, **167**, 222.

SAPRIN, A. N., NAGLER, L. G., KOPERINA, YE. V., KRUGLYAKOVA, K. YE & EMANUEL, N. M. (1966*c*) Kinetics of Change in the Content of Free Radicals in the Blood and Organs of Mice with Experimental Leukaemia. II. *Biofizika*, **11**, 706.

SWARTZ, H. M., MAILER, C., AMBEGAONKAR, S., ANTHILINE, W. E., MCNELLIS, D. R. & SCHNELLER, S. J. (1973) Paramagnetic Changes during Development of a Transplanted AKR/J Leukemia in Mice as Measured by Electron Spin Resonamce. *Cancer Res.*, **33**, 2588.